# Human-Derived Motor Neuron Progenitor Cell Implantation Rescues Motor Endplates Following Peripheral Nerve Injury

**DOI:** 10.1016/j.jhsg.2026.101161

**Published:** 2026-10-12

**Authors:** Luigi P. Gonzales, Chris Lee, Arjun T. Gupta, David J. Wright, Tyler R. Johnston, Sang Ho Yoon, Robert Hunt, Oswald Steward, Ranjan Gupta

**Affiliations:** ∗ Department of Orthopedic Surgery, University of California, Irvine, CA; † Department of Anatomy & Neurobiology, University of California, Irvine, CA; ‡ Reeve-Irvine Research Center, University of California, Irvine, CA

**Keywords:** Motor endplate, Motor neuron progenitors, Peripheral nerve injury, Stem cells

## Abstract

**Purpose:**

Degradation of motor endplates (MEPs) following peripheral nerve injury (PNI) is a major determinant of functional recovery, as prolonged denervation renders target muscles progressively unreceptive to reinnervation. While prior studies have demonstrated that stem cell–based therapies may provide neurotrophic support following PNI, their potential effects on MEP preservation have not been well studied. We hypothesized that transplantation of human-induced pluripotent stem cell (hiPSC)–derived motor neuron progenitors (MNPs) into chronically denervated muscle would be associated with preservation of MEP architecture.

**Methods:**

With Institutional Animal Care and Use Committee approval, chronic denervation injuries were created in mice via sciatic nerve transection. After 4 months of denervation, when MEPs have consistently degraded in this animal model, the denervated tibialis anterior (TA) was injected with low-dose MNPs (10,000 cells), high-dose MNPs (500,000 cells), or phosphate-buffered saline (negative control). Using a previously established method, hiPSCs were differentiated into functional MNPs prior to injection. At selected time points post-injection, the TA was harvested and immunostained to confirm MNP viability and detect the presence of MEPs.

**Results:**

Transplanted MNPs remained viable within host muscle at both evaluated time points. No identifiable MEPs were detected in phosphate-buffered saline-treated chronically denervated muscle. In contrast, MNP-treated muscles demonstrated the presence of MEP-like structures following prolonged denervation. Qualitative differences in MEP density and morphologic complexity were observed between treatment groups, with greater preservation seen in the higher-dose cohort. Contralateral uninjured muscles demonstrated intact native MEP architecture.

**Conclusions:**

Intramuscular transplantation of hiPSC-derived MNPs into chronically denervated muscle was associated with preservation of MEP morphology in this preclinical proof-of-concept model. These preliminary findings suggest that cellular therapies may represent a potential adjunctive strategy to preserve end-organ receptivity following delayed peripheral nerve injury.

**Type of study/level of evidence:**

Preclinical controlled animal study, Level V.

Peripheral nerve injuries (PNIs) often result in substantial morbidity and incomplete functional recovery despite modern surgical management. Current reconstructive strategies—including primary repair, autografting, allografting, nerve conduits, and nerve transfers—aim to restore axonal continuity and improve functional outcomes, but results remain variable and limited.^1^, ^2^, ^3^

One of the principal barriers to successful recovery following PNI is the progressive degeneration of the distal neuromuscular target during prolonged denervation. Although peripheral axons retain some regenerative capacity following Wallerian degeneration, axonal regeneration is often slow and may require growth over considerable distances before reaching the target muscle.^4^^,^^5^ During this interval, denervated skeletal muscle undergoes atrophy, and motor endplates (MEPs) progressively degenerate from complex mature “pretzel-like” structures into simplified plaque-like remnants before eventual disappearance.^3^^,^^6^

This degeneration has important clinical consequences. Even when successful axonal regeneration occurs, delayed reinnervation may fail to restore meaningful function because the target muscle and its neuromuscular junctions are no longer receptive to reinnervation.^6^^,^^7^ Accordingly, delayed surgical intervention is associated with substantially poorer outcomes.

For patients who present beyond the optimal treatment window, reconstructive options become increasingly limited. In select cases, palliative interventions such as tendon transfers or shoulder arthrodesis may provide partial functional compensation, but these procedures involve substantial tradeoffs and do not restore normal neuromuscular function.^8^, ^9^, ^10^ These limitations underscore the clinical importance of strategies aimed at preserving the distal motor apparatus following prolonged denervation.

In an ideal setting, definitive intervention would occur before irreversible degeneration of the neuromuscular target. In clinical practice, however, delayed presentation is common because of variability in injury recognition, referral patterns, diagnostic complexity, and associated trauma burden.^11^ Accordingly, interventions that preserve or restore MEP architecture may extend the therapeutic window for successful reconstruction.

Two broad biological strategies have been explored to address denervation-associated degeneration: trophic factor delivery and cellular therapies. While both approaches show promise, substantial challenges remain, including optimization of timing, dosage, delivery methods, and immunologic considerations.^12^, ^13^, ^14^

Human-induced pluripotent stem cell (hiPSC)–derived motor neuron progenitors (MNPs) represent a potentially attractive therapeutic platform because of their capacity to provide neurotrophic support and modulate the local denervated microenvironment. To date, however, the effects of intramuscular transplantation of hiPSC-derived MNPs on chronically denervated motor endplates have not been evaluated.

In this proof-of-concept study, we examined whether transplantation of hiPSC-derived MNPs into chronically denervated muscle would be associated with preservation of motor endplate morphology in a murine model of prolonged denervation. We hypothesized that hiPSC-derived MNP transplantation would preserve MEP architecture relative to untreated chronically denervated muscle.

## Materials and Methods

### Animals

All experiments were approved by our Institutional Animal Care and Use Committee (IACUC), in compliance with the National Institutes of Health and national laws concerning the care and use of laboratory animals. NOD SCID IL2R gamma null (NSG) mice were used to model chronic denervation of the tibialis anterior (TA) muscle, as they lack T and B lymphocytes and are routinely used for xenograft studies.^12^^,^^15^ TA muscles were injected with different numbers of MNPs and harvested at either four or six weeks post-injection (Table). The 4-week time point was selected to allow sufficient time for MNP survival and activity, whereas the 6-week time point assessed whether extended duration influenced MNP persistence and MEP morphology.

**Table:** Number of Muscle Samples for Each Injection Concentration and Harvest Time From Injection

Estimated MNP Count per Injection	Muscle Harvest Time From Injection	Number of Tibialis Anterior Muscles Harvested
0	4 weeks	2
	6 weeks	2
10,000	4 weeks	3
	6 weeks	3
500,000	4 weeks	4
	6 weeks	9

### Chronic denervation injury model

Mice were anesthetized via intraperitoneal (IP) injection of ketamine/xylazine (80–100 mg/kg and 5–10 mg/kg) with supplemental doses of one-third the initial dose as needed. Anesthesia was monitored by toe pinch, respirations, heart rate, mucous membrane color, and muscle relaxation level.

The surgical site was shaved and cleaned with Betadine (3X) and then rinsed with 70% ethanol. A 1.5-cm midline incision was made to expose hindlimb muscles overlying the sciatic nerve, and the nerve was exposed by blunt dissection with surgical forceps. The right sciatic nerve was transected, a 10-mm segment was excised, and the proximal stump was sutured outside the muscle to create a critical-size defect and prevent regeneration. The left sciatic nerve was mobilized and returned as a control. Muscles were closed with absorbable sutures, and the skin was closed with nylon sutures. The surgical procedure was performed within 30–45 minutes for each animal. Mice were recovered on a heating pad to prevent hypothermia, received buprenorphine and subcutaneous saline injections, and were monitored every 5 minutes until they regained consciousness.

### Human-induced pluripotent stem cell differentiation into motor neuron progenitor cells

hiPSCs were obtained from the Coriell Institute (CW60161; 14-year-old male donor). A single hiPSC line was used for all experiments. Genomic stability was monitored using qPCR-based karyotyping every ten passages, with no abnormalities detected. Additional high-resolution genomic analyses (eg, SNP array or whole-genome sequencing) were not performed. Pluripotency was confirmed through in vitro expansion, immunostaining for pluripotency markers (OCT4, NANOG, SSEA4, and SOX2), and in vivo transplantation assays demonstrating tumorigenesis and differentiation into all three germ layers.

hiPSCs were cultured on Matrigel (Corning, 354277)-coated plates in mTeSR1 medium (STEMCELL TECH, 85850) at 37 °C in 5% CO2 and were passaged weekly with ReLeSR (STEMCELL TECH, 100-0483). MNP differentiation followed the method previously described by Hu and Zhang.^16^ Cells were dissociated with Dispase (STEMCELL TECH, 07923) at 37 °C for 10 minutes; cell aggregates were transferred into a nontreated flask and cultured in medium containing Dulbecco's Modified Eagle Medium (mi)/F12 (Gibco; 11330057), 20% KnockOut Serum Replacement (Gibco; 10828028), 1X minimum essential medium (MEM) with nonessential amino acids (Gibco; 11140050), 1 mM L-glutamine (Gibco; 25030081), and 0.1 mM β-mercaptoethanol (Gibco; 21985023), with daily medium changes for the first 4 days. On day 4, the medium was replaced with neural induction (NI) medium containing DMEM/F12, 1X N2 supplement (Gibco, 17502048), MEM nonessential amino acids, and 2 μg/mL heparin (Sigma, H3149). The medium was changed on day 6. On day 7, embryoid bodies were seeded onto a laminin (Gibco, 23017015)-coated 6-well plate in NI medium and cultured at 37 °C in 5% CO2. On day 10, medium was replaced with NI medium supplemented with 0.1 μM retinoic acid (RA; Sigma-Aldrich, R2625). The medium was changed on days 12 and 14. On day 15, neural tube-like rosettes were collected and transferred into a nontreated flask, then cultured in NI medium supplemented with 1X B-27 supplement (Gibco, 12587010), 0.1 μM RA, and 1 μM purmorphamine (Pur, StemGent, 04-0009), with changes every other day. On day 28 of differentiation, neurospheres were collected and incubated with Accutase (Gibco, A1110501) at 37 °C for 5 minutes and dissociated into single cells. Cells were counted using an automated cell counter and then plated or used for transplantation. Cells plated onto coverslips were cultured in neural differentiation medium for 28 days. MNPs were confirmed by neuron-specific class III β-tubulin (TUJ1, BioLegend, cat no. 801201, 1:1000) and choline acetyltransferase (ChAT; Millipore, cat no. AB144P, 1:1000) immunostaining, labeled with appropriate secondary antibodies, and coverslipped with Fluoromount containing DAPI (Fig. 1).

**Figure 1:**
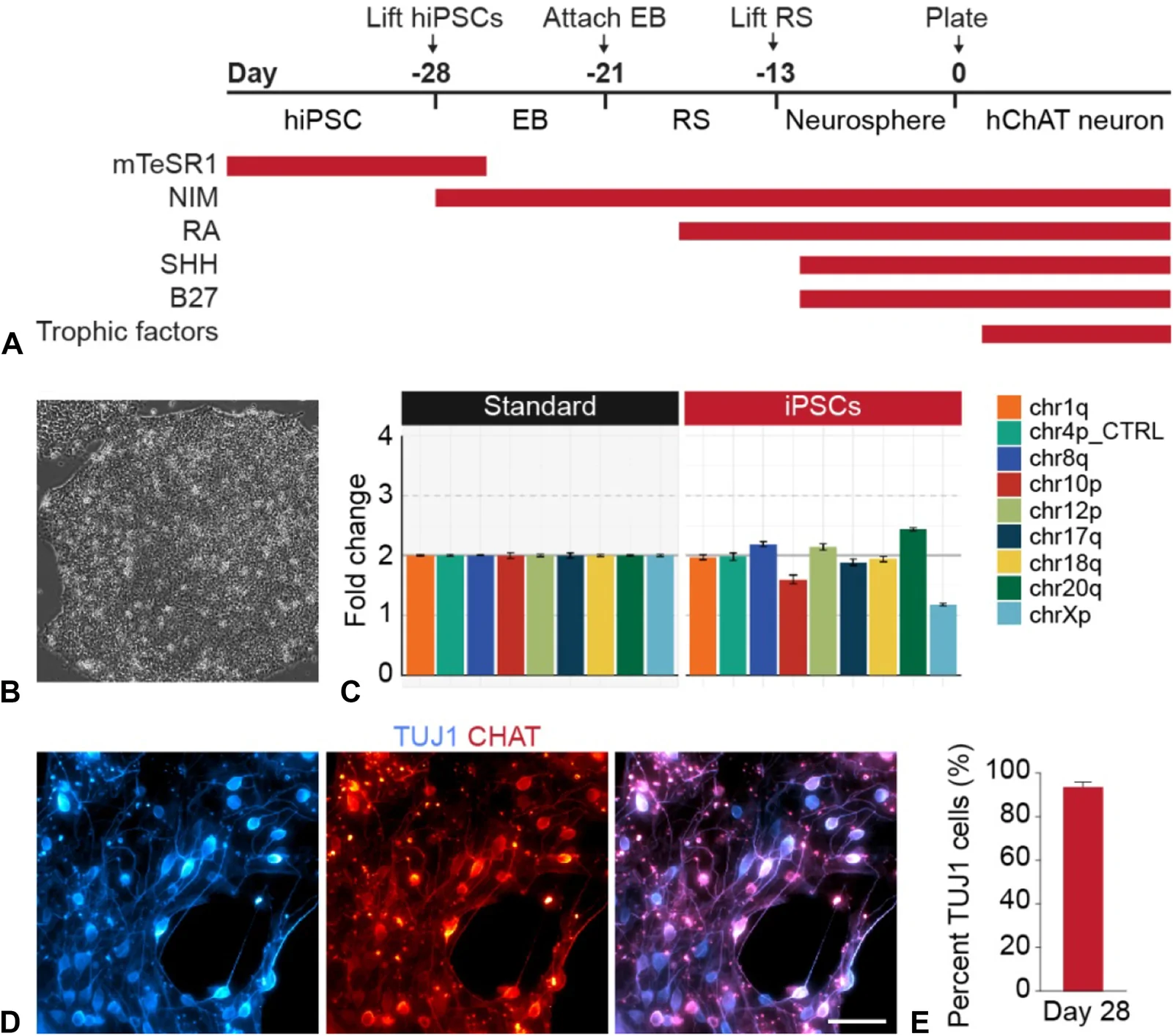
Confirmation of ChAT-positive motor neuron progenitors. **A.** Experimental timeline for generating MNPs from hiPSCs. **B.** Representative image of a hiPSC colony before differentiation. **C.** Real-time qPCR-based karyotyping shows normal characteristics of the hiPSC line used in these studies. **D.** hiPSC-derived motor neuron progenitors immunostained at 28 days in vitro for TUJ1 (blue) and ChAT (red). Scale bar, 50 μm. **E.** Quantification of ChAT-positive neurons at 28 days *in vitro*.

### Karyotyping and analysis of chromosomal abnormalities

Genomic DNA was extracted from iPSCs using a DNA purification kit (STEMCELL TECH, 79020). Karyotyping was performed with the hPSC Genetic Analysis Kit (STEMCELL TECH, 07550). A total of 10 ng of genomic DNA was loaded and analyzed by real-time qPCR. The comparative Ct method was used to analyze the data.

### Transplantation of motor neuron progenitors

Mice were transplanted with MNPs at 4 months post-injury (Fig. 2). Mice were restrained with the injured leg exposed. The injured anterior leg was washed with 70% ethanol, and the hair was combed to the side to expose the TA muscle. The muscle was palpated to confirm the correct location. MNPs were suspended in a defined culture medium (DMEM/F12 with 1X N2 supplement, 1X MEM nonessential amino acids, and 1 mg/mL heparin), consistent with previously described protocols.^16^ The solution was injected (1 mL, 26-gauge needle) into the proximal, middle, and distal portions of the TA muscle to promote broader distribution of transplanted cells and maximize exposure of the denervated muscle to trophic factors, as MEPs are spatially clustered.^17^ Following transplantation, the hindlimb was cleaned with 70% ethanol, and the mice were returned to their cage.

**Figure 2:**
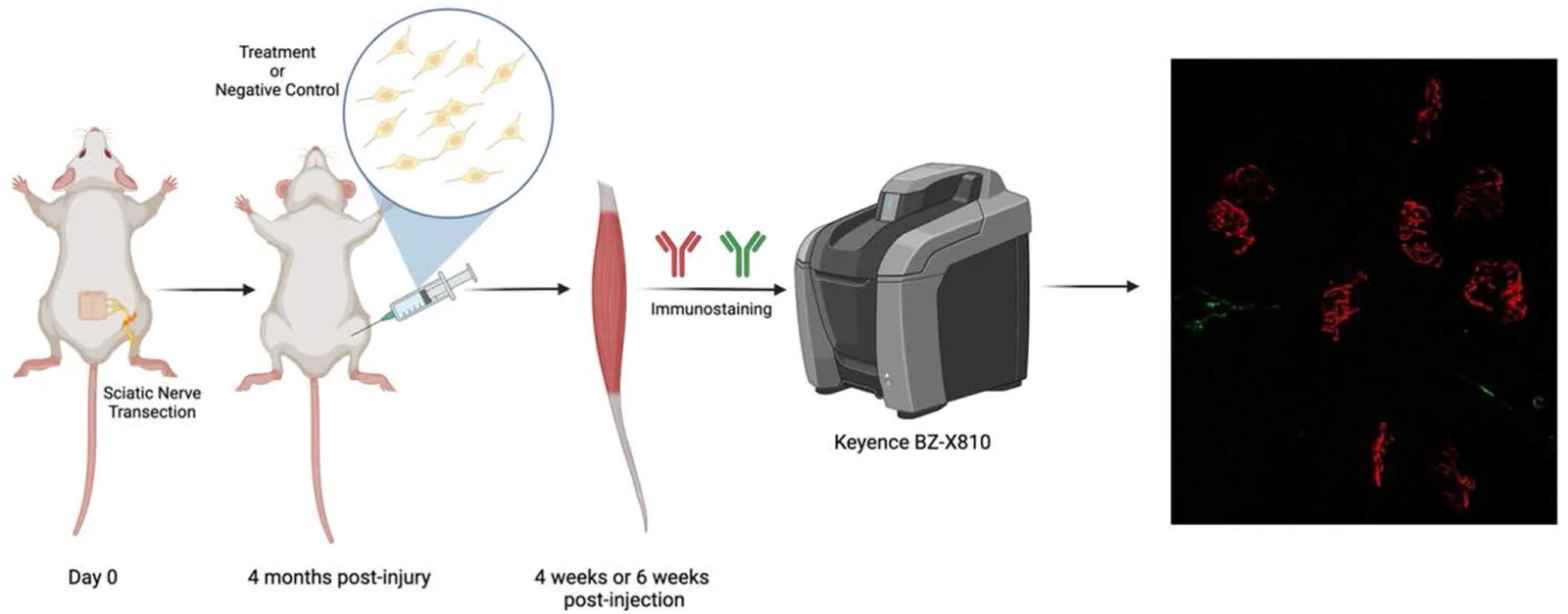
Experimental design and workflow. Mice underwent sciatic nerve transection (day 0) to induce chronic denervation. After 4 months, MNPs or PBS were injected into the tibialis anterior muscle. Muscles were harvested at 4 or 6 weeks post-injection, followed by immunostaining and fluorescence imaging (Keyence BZ-X810) to assess motor endplates (red) and neural elements (green).

### Tibialis anterior muscle harvesting

Mice were euthanized via cervical dislocation. Hindlimbs were sprayed with 70% ethanol to maintain a clean field and minimize contamination during subsequent dissection and tissue collection. The TA was exposed entirely to visualize the distal TA and extensor digitorum (ED) tendons. The TA and ED tendons were transected with microdissecting scissors, and a thin-tip tweezer was placed between the distal TA tendon and the ED tendon to separate the two muscles. The TA tendon was pulled upward until only the area below the knee was attached and was then cut below the knee.

### Muscle immunohistology

The harvested TA was washed with phosphate-buffered saline (PBS) and fixed in 4% paraformaldehyde (PFA) overnight. The following day, the muscle was washed with PBS 5 times for a duration of 5 minutes each, followed by washing with 0.1 M glycine for 30 minutes. The muscle was then placed in a solution of 1000 μL of normal donkey serum (NDS) (Jackson ImmunoResearch) in 10 mL 1% Triton X-100 blocking solution for 1 hour. The muscle was then incubated in a solution of primary antibodies diluted in blocking solution (anti-neurofilament antibody [BioLegend, cat no. 837904]; anti-synaptophysin antibody [Dako, cat no. M7315]; rabbit recombinant monoclonal Alas1 antibody [Abcam, cat no. 154860]) and antifade mounting medium with DAPI (Vector Laboratories, cat no. H1200) at 4 °C overnight with shaking.

The muscle was then washed with PBS thrice for 5 minutes each and incubated with 1:1000 secondary antibody (Alexa Fluor 488 donkey anti-mouse IgG [H+L]; Thermo Fisher, cat no. A21202) and 1:1000 α-bungarotoxin, Alexa Fluor 594 conjugate (Thermo Fisher, cat no. S11227) on a shaker at 4 °C overnight. Muscles were then washed thrice with PBS for 5 minutes each and mounted on a microscope slide.

### Motor neuron progenitor cell and motor endplate imaging

Samples were imaged using the Keyence BZ-X810 fluorescence microscope. Whole muscles were scanned in the X-, Y-, and Z-planes. All MNPs and MEPs were located and imaged at 20× with Z-stack pitch rates ranging from 0.5 to 0.8, depending on the depth needed to capture the full image in the Z-plane. The Keyence BZ-X810 Analyzer was used to perform haze reduction and black balance on the raw Z-stack images, and images were further optimized using ImageJ.

### Outcome measurement

MNP cell survival was confirmed by the presence of overlapping immunohistological signals between the human anti-mitochondrial and anti-nuclear DNA antibodies. MEP morphology (pretzel, intermediate, plaque) was assessed according to established criteria defined by Marques et al.^18^ The pretzel morphology was defined as MEPs with high complexity and multiple perforations within the boundaries of the MEP.^18^ Intermediate morphology was described as MEPs with a clear but incomplete outline and no perforations.^18^ Plaque morphology was considered a sign of unhealthy MEPs and typically had minimal visibility and a lack of structure.^18^ Innervation status of the MEPs (MEPs with visualized nerve terminals vs MEPs without visualized nerve terminals) was determined by the presence or absence of immunostaining for neurofilament and synaptophysin within MEP Z-stacks.

## Results

### Survival of transplanted hiPSC-derived motor neuron progenitors

Colocalization of human anti-nuclear and anti-mitochondrial antibody immunofluorescence was observed in TA muscles of mice that received MNP transplants (Fig. 3), confirming MNP presence and viability. No specific human-derived immunofluorescence signal for either antibody was detected in the control TA muscle, supporting the absence of systemic migration of MNPs. These findings were consistent at both 4 and 6 weeks post-transplantation, indicating that the MNPs can survive in the host tissue for at least 6 weeks.

**Figure 3:**
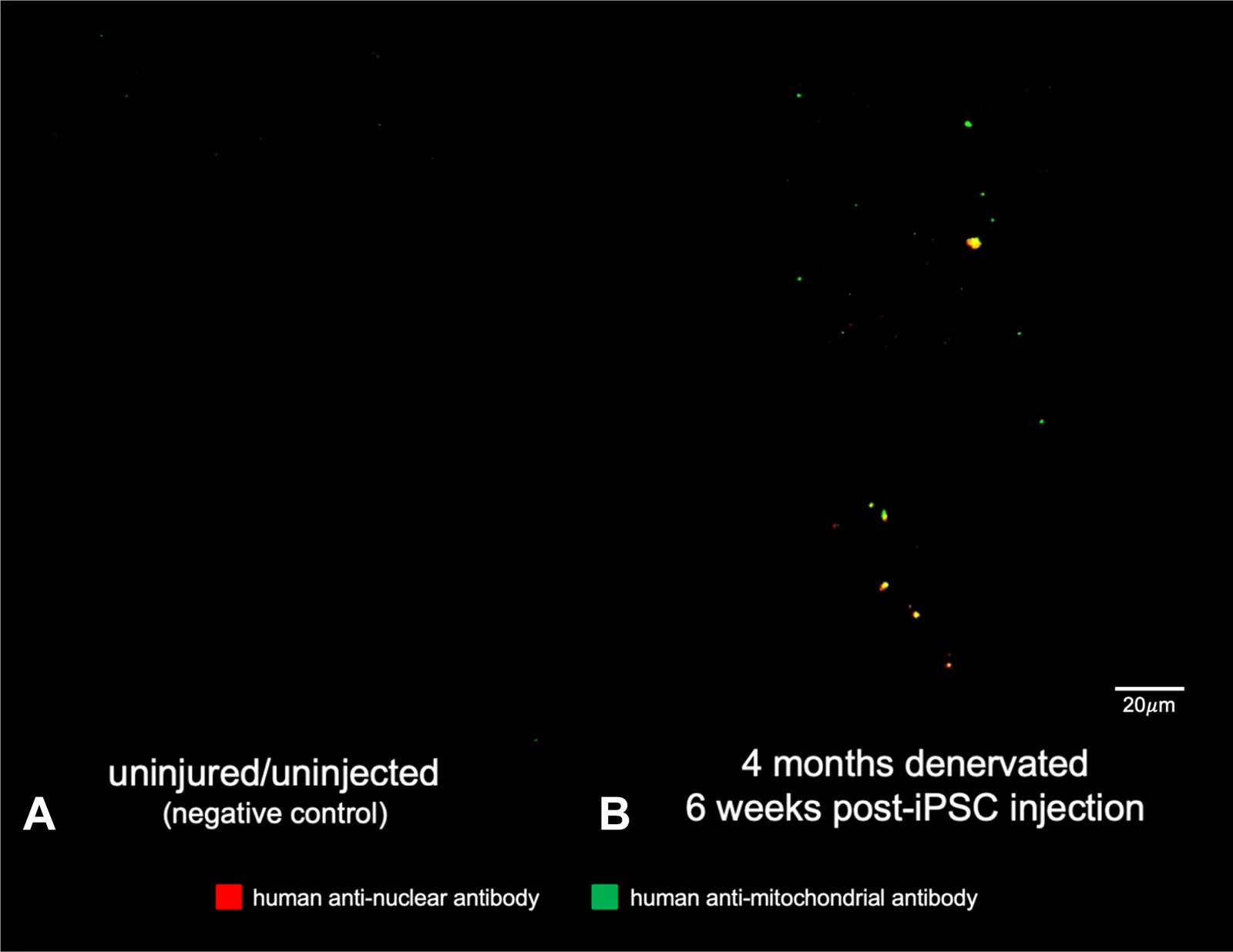
**A** No specific human-derived signals were detected in the negative control TA muscle, which did not receive MNP transplantation. **B.** Confirmation of transplanted MNP survival in mouse TA muscle 6 weeks post-injection. MNPs were confirmed by visualizing the overlay between the human anti-nuclear antibody (red) and human anti-mitochondrial antibody (green).

### Preservation of motor endplates following motor neuron progenitor transplantation

MEPs were not detected in the negative control TA muscles after 4 months of denervation. In contrast, MEPs were present in MNP-treated muscles despite prolonged denervation. The detected MEPs were often fragmented and did not exhibit as clear a morphology as those from the uninjured healthy control muscle (Fig. 4).

**Figure 4:**
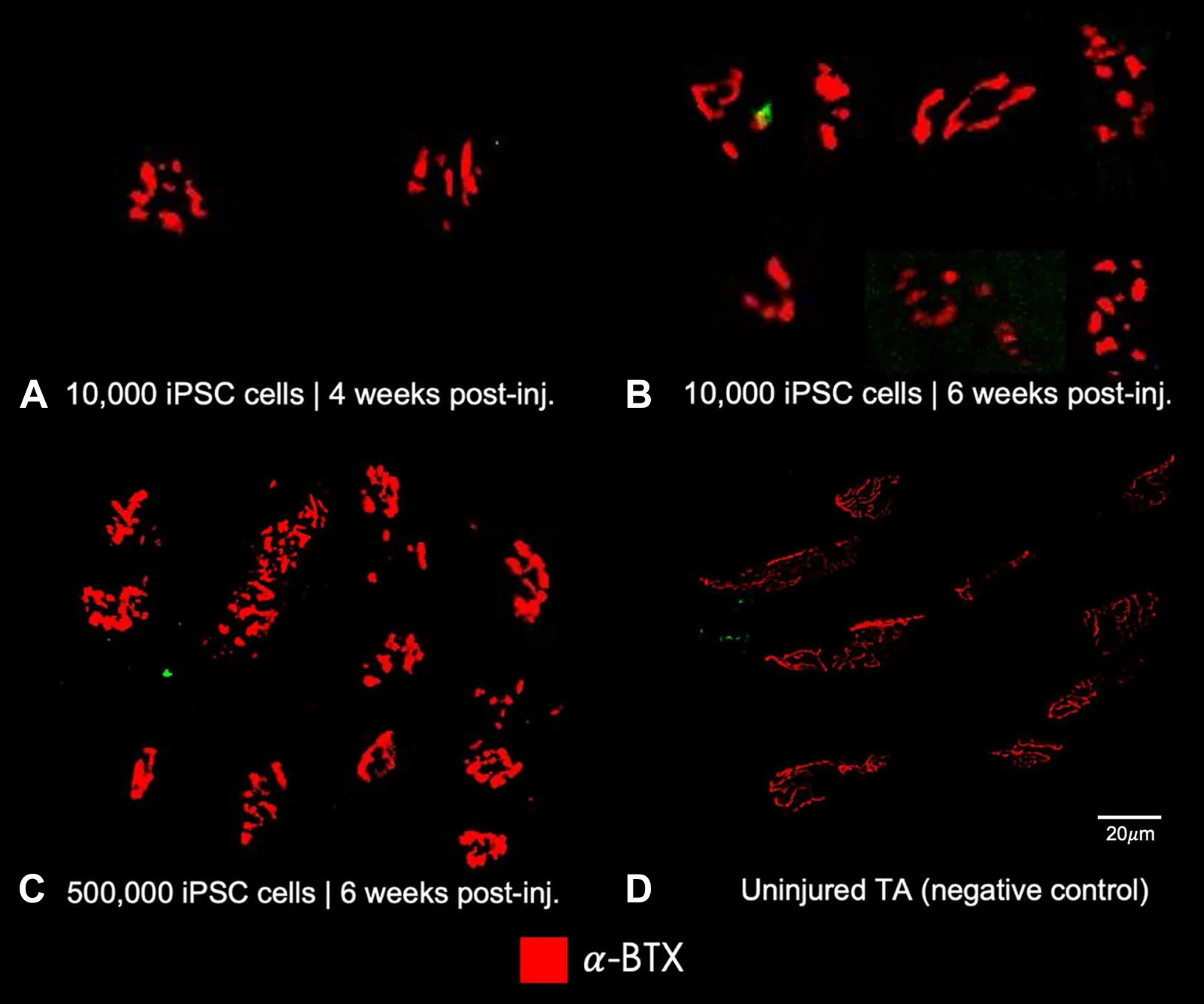
MEPs of 4-month-denervated mouse TA muscles at different time points after injection and MNP injection doses. **A.** Minimal number of MEPs with plaque/intermediate morphology in mice injected with low-dose iPSCs 4 weeks post-injection. **B.** Increased number of MEPs with plaque/intermediate morphology with low-dose iPSCs 6 weeks post-injection. **C.** Intermediate/pretzel MEPs from samples injected with high-dose iPSCs 6 weeks post-injection. **D.** MEPs from uninjured mouse TA muscle.

### Preservation of motor endplate structures following MNP transplantation

MEP number and complexity in different muscle samples qualitatively appeared to vary with MNP dose. In the 10,000-MNP dose group, few MEPs were detected. Furthermore, MEPs that were present were very small and had minimal fluorescence signal. In the 500,000-MNP dose group, a greater number of MEPs with larger size and complexity were observed (Fig. 4). These observations are qualitative, and no statistically significant differences in MEP number or morphology were identified between dosing groups or between muscle samples that were harvested 4 or 6 weeks post-transplantation for either MNP injection concentration.

## Discussion

Functional recovery following peripheral nerve injury remains limited despite advances in reconstructive techniques, particularly in cases involving delayed presentation and prolonged denervation. The progressive degeneration of the distal neuromuscular apparatus, including motor endplate loss, remains a major biological barrier to successful recovery.^3^^,^^19^

Multiple prior studies have investigated stem cell–based strategies to improve outcomes following peripheral nerve injury. Mesenchymal stem cells, adipose-derived stem cells, and other progenitor populations have demonstrated regenerative or trophic effects in preclinical models.^12^^,^^14^^,^^20^^,^^21^ However, the specific effects of hiPSC-derived motor neuron progenitors (MNPs) on chronically denervated motor endplate architecture have not previously been described.

In this proof-of-concept study, intramuscular transplantation of hiPSC-derived MNPs into chronically denervated muscle was associated with preservation of MEP-like structures in a model where untreated chronically denervated muscle demonstrated complete absence of identifiable motor endplates. While the observed structures in transplanted muscle did not demonstrate the organization or complexity of native uninjured motor endplates, their presence following prolonged denervation suggests that the local biologic environment may have been altered in a manner supportive of motor endplate preservation. Qualitative differences between treatment groups were also observed, with higher-dose transplantation associated with greater apparent structural preservation. However, because formal quantitative morphometric analysis was not performed, these observations should be interpreted cautiously and viewed as hypothesis-generating rather than definitive evidence of dose dependence.

The mechanism underlying these findings remains uncertain. One plausible explanation is that transplanted hiPSC-derived MNPs provide local trophic or paracrine support that modifies the chronically denervated muscle microenvironment in a manner favorable to preservation of neuromuscular architecture. Prior studies have demonstrated that motor neuron progenitor populations may secrete neurotrophic factors relevant to neuronal survival and neuromuscular maintenance.^22^, ^23^, ^24^ However, the present study was not designed to establish mechanistic causality, and any mechanistic interpretation remains speculative.

From a translational perspective, the concept of preserving the distal neuromuscular target during periods of delayed reconstruction is clinically relevant. In scenarios such as complex trauma, brachial plexus injury, or delayed referral, preservation of end-organ receptivity could theoretically improve the effectiveness of later reconstructive intervention. However, this study does not establish functional recovery, successful reinnervation, or clinical therapeutic efficacy. Hence, these findings should be viewed as preliminary biologic evidence supporting further investigation into whether cellular therapies may help preserve the denervated neuromuscular environment.

Several limitations must be acknowledged. First, this was a proof-of-concept pilot study with a limited sample size, constraining both statistical interpretation and generalizability.

Second, although transplanted human-derived cells remained detectable within host muscle tissue, the study did not assess functional outcomes such as electrophysiologic recovery, contractile strength, behavioral recovery, or reinnervation following delayed nerve reconstruction. Accordingly, preservation of MEP-like morphology cannot be assumed to reflect functional neuromuscular recovery. Third, outcome assessment relied primarily on qualitative histologic evaluation rather than formal quantitative morphometric analysis. While structural differences between groups were visually apparent, quantitative assessment of MEP density, complexity, and architecture would strengthen future studies. Fourth, the precise origin and biologic identity of preserved MEP-like structures cannot be fully characterized in the present model, particularly given limitations in available species-specific presynaptic markers.

Fifth, the study was not designed to define the mechanism of action. Whether observed effects reflect trophic signaling, paracrine support, altered muscle microenvironment, or other biologic pathways remains unknown. Finally, although hiPSC-derived cellular therapies offer conceptual translational advantages, additional work will be required to address scalability, reproducibility, genomic stability, safety, and clinical feasibility.

This proof-of-concept preclinical study demonstrates that intramuscular transplantation of hiPSC-derived motor neuron progenitors into chronically denervated muscle was associated with preservation of MEP-like structures in a murine model of prolonged denervation, in contrast to untreated chronically denervated controls in which no identifiable motor endplates were observed. Although these findings do not establish functional recovery or therapeutic efficacy, they provide early biologic evidence that cellular therapies may help preserve components of the distal neuromuscular target during prolonged denervation. Given that degeneration of the neuromuscular target remains a major barrier to successful delayed peripheral nerve reconstruction, this concept has important translational relevance. Further quantitative, mechanistic, and functional studies are required to determine the therapeutic potential of this strategy.

## Ethics declaration

Written informed consent to take part in the study and to publish the article has been obtained from all participants or their legal representatives. The privacy rights of participants have been observed.

This study included organ or tissue donors. This study includes human biological material and consent was obtained by donors, or their next of kin or legal representatives, for use in this study and for publication of the article. The samples used in this research were not sourced from executed prisoners or prisoners of conscience.

This study was performed in compliance with relevant laws, regulatory frameworks and guidelines where the research took place. This study was approved by the University of California, Irvine. (Approval No. #2026)

## Conflicts of Interest

No benefits in any form have been received or will be received related directly to this article.
